# Echocardiographic diagnosis of rare pathological patterns of sinus of Valsalva aneurysm

**DOI:** 10.1371/journal.pone.0173122

**Published:** 2017-03-14

**Authors:** Yali Yang, Li Zhang, Xinfang Wang, Qing Lü, Lin He, Jing Wang, Bin Wang, Ling Li, Li Yuan, Jinfeng Liu, Shuping Ge, Mingxing Xie

**Affiliations:** 1 Department of Ultrasound, Union Hospital, Tongji Medical College, Huazhong University of Science and Technology; Hubei Provincial Key Laboratory of Molecular Imaging, Wuhan, Hubei, China; 2 The Heart Center, St. Christopher's Hospital for Children and Drexel University College of Medicine, Philadelphia, Pennsylvania, United States of America; University of Cincinnati College of Medicine, UNITED STATES

## Abstract

**Objective:**

To evaluate the value and improve the diagnostic accuracy of echocardiography in the diagnosis of a sinus of Valsalva aneurysm (SVA) with rare pathological patterns.

**Methods:**

Echocardiographic features and surgical findings from 270 Chinese patients with SVA treated in the last 18 years (1995–2013) at the Union Hospital were compared retrospectively; 22 of 270 cases had rare patterns.

**Results:**

The patients with SVA, a rare origin, a rare extending position, or a rare course accounted for 3.4%, 7.4%, and 0.4% of the 270 cases, respectively. The three most common aneurysmal complications of the patients with rare patterns were severe aortic regurgitation (16), obstruction of the ventricular outflow tract or valvular orifice (3), and conduction disturbance (3). The origin, course, extending position and rupture status of the SVAs determined by echocardiography were entirely consistent with surgical findings in 81.8% of the 22 cases. With the exception of one failed diagnosis of an aneurysmal wall dissection and one misdiagnosis of a descending aortic dissection, the echocardiographic results of SVA complications and associated cardiovascular lesions were also confirmed.

**Conclusion:**

We could accurately diagnose SVAs with rare pathological patterns by echocardiographic identification of distinguishing features. However, for several conditions, we could not accurately identify the origin or course of the aneurysm or define its relationship to adjacent structures using conventional echocardiography alone. Therefore, we recommend combining conventional echocardiography with different imaging techniques, such as transesophageal echocardiography, three-dimensional echocardiography, computed tomography angiography, and aortic angiography.

## Introduction

A sinus of Valsalva aneurysm (SVA) is an uncommon congenital malformation. The prevalence of SVA in the Chinese population is 1.2% to 1.8% and is 0.14% to 0.96% in Western populations with congenital heart disease [1,2]. It is well known that SVAs arise mostly from the right and non-coronary sinuses and rarely from the left coronary sinus (LCS) or multiple sinuses. When SVA ruptures, it is more apt to enter the right ventricle (RV), followed by the right atrium (RA). In a few cases, they extend into multiple chambers simultaneously or take up uncommon positions such as the left ventricle (LV), left atrium (LA), interventricular septum, interatrial septum, superior vena cava, pulmonary artery, and pericardial or pleural cavities.

Echocardiography is currently the diagnostic modality of choice for the evaluation of SVAs [3]. However, articles describing the role of ultrasound in diagnosing the rare pathological patterns are mostly case reports, and details surrounding the use of ultrasound are seldom provided. Operator inexperience makes it easy to misdiagnose or to fail to diagnose SVAs. In this paper, we retrospectively compare the echocardiographic features and surgical findings of SVA patients with rare patterns to evaluate the diagnostic value of echocardiography and discuss how to improve the diagnostic accuracy.

## Materials and methods

### Patient population

From June 1995 to August 2013, 270 patients with congenital SVAs underwent surgical repair at our hospital, comprising 2.1% of 13,061 open-heart operations. The ratio of male to female patients was approximately 2 to 1 (181:89). The age ranged from 4 months to 67 years (mean 28.4 ± 13.0 years). Presenting symptoms included dyspnea, easy fatigability, palpitations, and chest pain.

Based on the World Health Organization’s definition of a rare disease (0.65‰–1‰ of the total population) and the incidence of SVAs in the Chinese population, a rare pattern was defined as a pattern that occurred in less than 5% of patients with SVA. Based on the data available [1–3], the following pathological patterns were considered rare:

1. Multiple aneurysms originating from different sinuses
2. Aneurysm arising from the LCS
3. Aneurysm extending into the LA or LV
4. Aneurysm dissecting into the interatrial septum, interventricular septum, or free ventricular wall
5. Aneurysm extending into the superior vena cava
6. Aneurysm extending into the pulmonary artery
7. Extracardiac aneurysm
8. Same aneurysm extending into multiple cardiac chambers
9. Aneurysm with tortuous course

This study was approved by Ethics Committee of Tongji Medical College, Huazhong University of Science and Technology (IORG No: IORG0003571). All participants provided written informed consent. No experimental interventions were performed.

### Echocardiography

All patients underwent a complete echocardiographic examination using an ultrasound system (GE Vivid 7, Vingmed, Horten, Norway/GE Vivid 7 Dimension, Vingmed, Horten, Norway/Philips IE 33, Andover, MA, USA/Acuson Sequoia C256, Mountain View, CA, USA/HDI 5000, Agilent Technologies, Andover, MA, USA). Two-dimensional (2D) and three-dimensional (3D) transthoracic echocardiographic (TTE) scans were performed with a 2–7.5 MHz and 1–3 MHz probe, respectively, whereas 2D and 3D transesophageal echocardiographic (TEE) scans were performed with a 2–7 MHz probe.

The key planes included parasternal long-axis view of the left ventricle, short-axis view of the aortic root, long-axis view of the right ventricular outflow tract, apical 5-chamber view, and some modified, nonstandard views showing the aortic root. In particular, the following echocardiographic details were focused on when we reviewed the images of the rare patterns and compared them retrospectively with the operative results: (1)the origin, course, and terminating position of the aneurysm; (2) the continuity of the aneurysmal wall and its attachment (e.g., vegetation, thrombus); (3) the morphological change and movement during each cardiac cycle; (4)the compression or obstruction caused by a space-occupying effect of the aneurysmal body; (5) the severity of any involved valvular insufficiency, especially the aortic and tricuspid valves; (6) the twist or obstruction of the orifice and the compression of the adjacent coronary artery when observing the signs of myocardial ischemia; (7) arrhythmias, especially conduction abnormalities. The flow characteristics of the SVA and its associated lesions were also evaluated by color and spectral Doppler imaging.

## Results

### Distribution of SVAs with rare patterns

The distributions of the aneurysmal origin, the protruding position, and the course of 270 SVA patients with surgical repair are listed in Tables 1–3, respectively. A total of 22 patients had rare patterns. We found each of the previously mentioned rare patterns except protrusion into the superior vena cava; the rare patterns comprised 0.4%–3.3% of all SVA repairs. The most common aneurysmal complications were severe aortic regurgitation (72.7%, n = 16), obstruction due to space-occupying effect (13.6%, n = 3), and conduction disturbance (13.6%, n = 3). The most common associated cardiovascular abnormalities were aortic valvular malformation (n = 4) and ventricular septal defect (n = 2).

**Table 1:** Distribution of origins of SVAs in 270 patients.

Origin		Cases	Ratio (%)	Total Ratio (%)
**Common**	**RCS**	212	78.5	96.6
	**NCS**	49	18.1	
**Rare**	**Multiple**	7^a^	2.6	3.4
	**LCS**	2	0.7	
**Total**		270	100	100

^a^All sinuses in 6 cases; both right and non-coronary sinuses in 1 case were involved, respectively.

LCS: left coronary sinus; NCS: non-coronary sinus; RCS: right coronary sinus; SVA: sinus of Valsalva aneurysm

**Table 2:** Distribution of protruding positions of SVAs in 270 patients.

Protruding Position		Cases	Ratio (%)	Total Ratio (%)
**Common**	**RV**	185	68.5	92.6
	**RA**	65	24.1	
**Rare**	**Extracardiac**	9	3.3	7.4
	**LV**	4	1.5	
	**RA+RV**	3	1.1	
	**LA**	1	0.4	
	**IVS**	1	0.4	
	**IAS**	1	0.4	
	**PA**	1	0.4	
**Total**		270	100	100

IAS: interatrial septum; IVS: interventricular septum; LA: left atrium; LV: left ventricle; PA: pulmonary artery; RA: right atrium; RV: right ventricle

**Table 3:** Distribution of courses of SVAs in 270 patients.

Course	Cases	Ratio (%)	Total Ratio (%)
**Common (directly)**	269	99.6	99.6
**Rare (tortuously)**	1	0.4	0.4
**Total**	270	100	100

### Accuracy of echocardiography in diagnosing rare patterns

Surgical repair was undertaken based solely on the echocardiographically based diagnosis in 22 rare-pattern SVAs. Comparisons of the ultrasonic and surgical results are listed chronologically in Table 4. Only one small extracardiac aneurysm from the LCS was misdiagnosed as a diverticulum of the LCS. In the 13,061 open-heart operations, one ruptured peri-aortic abscess was misdiagnosed as SVA rupturing into left ventricular outflow tract. The sensitivity, specificity and accuracy of echocardiography in the diagnosis of rare pattern SVAs were 95.5%, 99.9% and 99.9%, respectively (Table 5). In addition, of 21 cases diagnosed by echocardiography before surgery, one aneurysm extending into both the RA and the RV but only rupturing into the RA was diagnosed as rupturing into the RA; one aneurysm from the right coronary sinus (RCS) with a tortuous course was misdiagnosed as an aneurysm from the non-coronary sinus (NCS) rupturing directly into the RV; one aneurysm from the RCS rupturing into the LV was misdiagnosed as having an intact aneurysmal wall due to the extremely small defect.

**Table 4:** Comparison of echocardiographic and operative results in patients with SVAs exhibiting rare patterns.

No.	Sex	Age, years	Combined echo- technique	Echocardiographic Results			Operative Findings
				SVA Pattern	SVA Complication	Associated Cardiac Anomalies	
1	M	21	–	Aneurysms of RCS and NCS protruding into RV	–	RVOT stenosis, VSD	Same
2	M	35	–	Aneurysm of RCS rupturing into RA	–	–	Aneurysm of RCS protruding into both RA and RV, but rupturing into RA
3	M	28	–	Aneurysm of RCS rupturing into PA	–	–	Same
4	M	44	3D TTE	Common dilation of all sinuses	AI	–	Dissection of aneurysmal wall, others same
5	M	51	–	Common dilation of all sinuses	AI	–	Same
6	M	27	–	Aneurysm of RCS rupturing into both RA and RV	RBBB, AP+AI, TV vegetation +TI	B`AV	Same
7	M	35	–	Aneurysm of NCS rupturing into RV	–	–	Aneurysm of RCS extending into RA, then going through and blocking tricuspid valve, rupturing into RV
8	M	15	3D TTE	Common dilation of all sinuses	AI	MP+MI, atrial septal aneurysm	Same
9	M	42	–	Common dilation of all sinuses	AI	Descending aortic dissection	Absent descending aortic dissection, others same
10	F	39	2D/3D TEE	Aneurysm of NCS rupturing into LV	Obstruction of LVOT, AP+AI	MI	Same
11	M	31	3D TTE	Aneurysm of LCS rupturing into LV	AP+AI	Dysplastic left coronary valve	Same
12	F	54	–	Unruptured aneurysm of NCS dissecting into IAS	AI	BAV+AS	Same
13	M	30	–	Ruptured aneurysm of RCS dissecting into IVS	LBBB. Obstructions of both LVOT and RVOT, AI	–	Same
14	F	48	–	Aneurysm of RCS extending into both RA and RV, but rupturing into RV	AP+AI	–	Same
15	M	29	–	Aneurysm of NCS rupturing into LA	Atrial fibrillation, AV vegetation +AP+AI	BAV	Same
16	F	23	2D/3D TEE	Aneurysm of RCS protruding into LV	AP+AI	–	Ruptured aneurysm, others same
17	M	25	–	Common dilation of all sinuses	AI	–	Same
18	F	57	3D TTE	Extracardiac aneurysm of NCS	Compression of LA and RA	–	Same
19	M	61	–	Common dilation of all sinuses	AP+AI	–	Same
20	M	0.28 (4 months)	–	Diverticulum of LCS	–	Small coronary-pulmonary artery fistula	Extracardiac aneurysm of LCS, others same
21	M	29	–	Aneurysm of RCS rupturing into LV in diastolic and RV in systolic via VSD	AP+AI	VSD, PFO	Same
22	F	42	–	Extracardiac aneurysm of NCS	Compression of RA, Aneurysmal wall calcification, AP+AI	–	Same

AI: aortic insufficiency; AP: prolapsed aortic valve; AS: stenosis of aortic valve; AV: aortic valve; AVR: aortic valve replacement; BAV: bicuspid aortic valve; IAS: interatrial septum; IVS: interventricular septum; LA: left atrium; LBBB: left bundle branch block; LCS: left coronary sinus LV: left ventricle; LVOT: left ventricular outflow tract; MI: mitral insufficiency; MP: prolapsed mitral valve; MVP: mitral valvuloplasty; MVR: mitral valve replacement; NCS: non-coronary sinus; PA: pulmonary artery; PFO: patent foramen ovale; RA: right atrium; RCS: right coronary sinus; RV: right ventricle; RBBB: right bundle branch block; RVOT: right ventricular outflow tract; SVA: sinus of Valsalva aneurysm; TEE: transesophageal echocardiography; TI: tricuspid insufficiency; TTE: transthoracic echocardiography; TV: tricuspid valve; TVP: tricuspid valvuloplasty; VSD: ventricular septal defect.

**Table 5:** Accuracy of echocardiography in patients with SVAs exhibiting rare patterns.

		Echocardiography		total
		+	−	
**surgery**	**+**	21	1	22
	**−**	1	13038	13039
**total**		22	13039	13061

The echocardiographic results of the SVA complications and the associated cardiovascular lesions were confirmed from the operation, except for one failed diagnosis of an aneurysmal wall dissection and one misdiagnosis of a descending aortic dissection.

**Fig 1:**
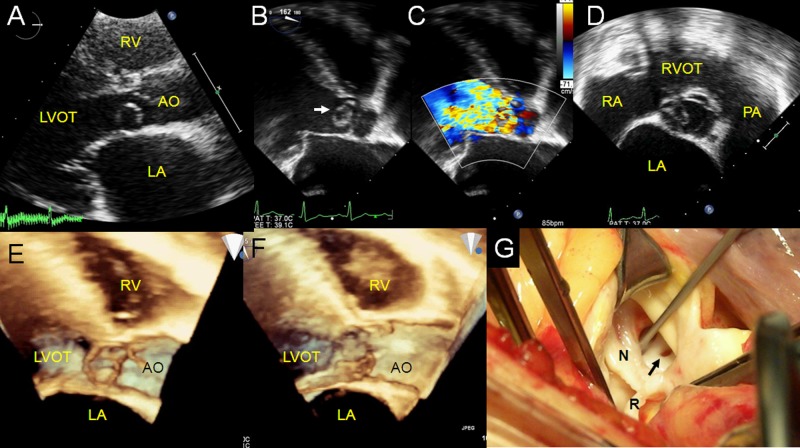
An aneurysm of the non-coronary sinus of Valsalva that has ruptured into the left ventricle. A saccular lesion extends into the left ventricular outflow tract, but we could not determine from transthoracic echocardiographic (TTE) scans whether it had ruptured or not (A). Transesophageal echocardiography (TEE) reveals a small aneurysmal defect (arrow) (B), the flows from the defect, the aortic valve (C), and the aneurysmal origin (D). We used real-time 3D TEE to further characterize the steric morphological changes during the cardiac cycles (E, F). An intraoperative photograph shows that the aneurysm (indicated by the probe in the middle of the picture) communicated with the left ventricle through the defect at its tip (arrow), viewed from the aortic root (G). AO: aorta; LA: left atrium; LVOT: left ventricular outflow tract; N: non-coronary cusp; PA: pulmonary artery; R: right coronary cusp; RA: right atrium; RV: right ventricle; RVOT: right ventricular outflow tract.

We made a diagnosis from the combination of transthoracic echocardiography with 3D TTE in 4 cases or with 2D/3D TEE images in 2 cases before surgery (Table 4). 3D echocardiography was superior to conventional ultrasound in showing the morphology of the aneurysm and its relationship with the adjacent structures, such as the aortic valve. Furthermore, a small defect that was unconfirmed or missed using conventional echocardiography was identified by both 2D and 3D TEE or 3D TTE in 2 cases (Fig 1).

### Echocardiographic features of different rare patterns

Similar to what was observed with common patterns, rare patterns also presented a thin-walled saccular or petaline lesion arising from the aortic root in continuation with the aortic annulus, with significant morphological changes and movement when rupturing or protruding into a cardiac chamber or vessel. If the aneurysm was intact, a vortex flow in it was revealed by color Doppler flow imaging (CDFI). If ruptured, a colorful continuous shunt, primarily during diastole, at the aneurysmal defect was seen, except when the SVA ruptured into the LV, in which case the shunt occurred only during diastole.

**Fig 2:**
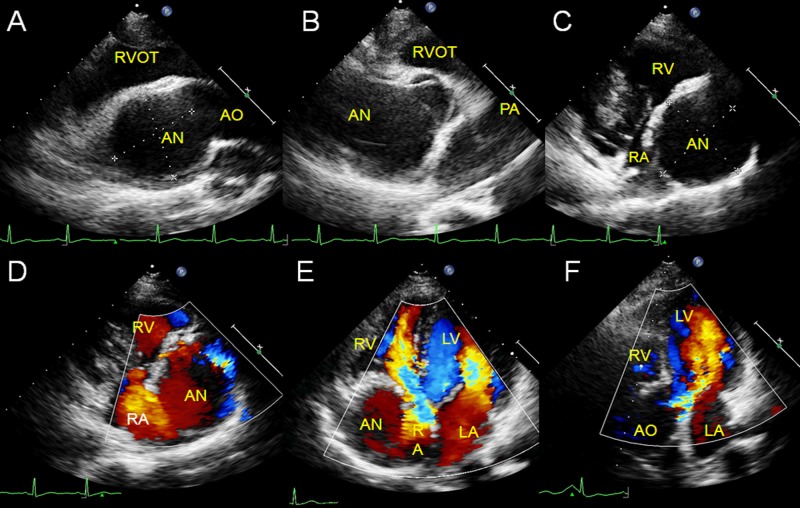
Huge extracardiac SVA compressing the right atrium. A huge aneurysm arising from the aortic root extended toward the right and posteriorly (A). It originated from the non-coronary aortic sinus revealed by the short-axis view of the aortic root (B). Both the right ventricular inflow tract (C, D) view and the apex four-chamber view (E) show compression of the right atrium and the vortex flow in the aneurysm. Severe aortic regurgitation occurred (F). AN: aneurysm; AO: aorta; LA: left atrium; LV: left ventricle; PA: pulmonary artery; RA: right atrium; RV: right ventricle; RVOT: right ventricular outflow tract.

**Fig 3:**
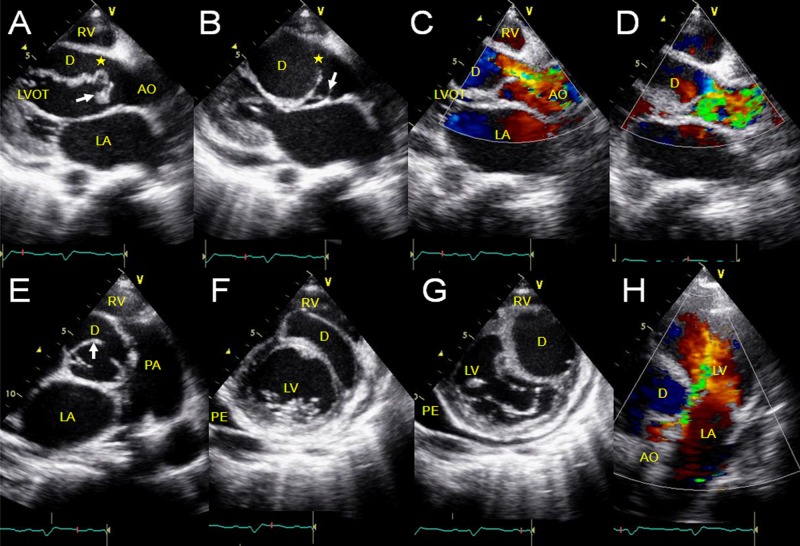
Aneurysm of the right sinus of Valsalva dissecting into the interventricular septum. The long-axis view of the left ventricle shows the aneurysm (arrow) extending into the interventricular septum and communicating with it through the aneurysmal defect (★), changing with systolic collapse (A) and diastolic expansion (B). CDFI reveals the to-and-fro flow at the site of the perforation of the aneurysm (C, D). The short-axis view of the aortic root shows that the aneurysm (arrow) originated from the right coronary sinus and communicated with the dissection (E). The left ventricular short-axis view shows the characteristic change in the area of the myocardial dissection and obstruction of the right ventricular outflow tract during diastole (F, G). The apex five-chamber view shows severe aortic regurgitation (H). AO: aorta; D: dissection; LA: left atrium; LV: left ventricle; LVOT: left ventricular outflow tract; PA: pulmonary artery; PE: pericardial effusion; RV: right ventricle

On the other hand, different rare patterns had some unique characteristics related mainly to the protruding position and the rupture status:

1. In 4 cases that extended into the LV, the saccular lesion arose between the sinus base and the aortic annulus. In 3 cases with an intact interventricular septum, the aneurysm was observed going back and forth between the aortic root and the left ventricular cavity (Fig 1). In another special case complicated by a huge subarterial ventricular septal defect, the aneurysm went back and forth between the LV and the RV through the septal defect, with the continuous shunt into the RV and the diastolic shunt into the LV related to the position of the aneurysmal defects. In all 4 cases, the adjacent coronary cusp and annulus prolapsed into the left ventricular outflow tract resulting in aortic regurgitation.
2. Extracardiac SVAs did not show significant motility or morphological changes during the cardiac cycle. In 6 cases in which the 3 sinuses were involved, the aortic sinuses had diffusely expanded aneurysms with a huge body but no evidence of compression. In 3 cases with a single aneurysm, 2 had a huge cavity that compressed the adjacent atrium or atriums (Fig 2).
3. In 3 cases with the same aneurysm extending into both the RA and the RV, each aneurysm originated from a position near or at the tricuspid annulus and the sac fused with the tricuspid valve to varying degrees. Compression and displacement of the tricuspid valve and subsequent valvular insufficiency were also present.
4. In the case in which the aneurysm dissected the interventricular septum, it extended into a cystic space in the upper part of the interventricular septum and communicated with it through the aneurysmal defect. The area of the dark space changed with the characteristic systolic collapse and diastolic expansion, resulting in obstruction of both ventricular outflow tracts. CDFI showed a to-and-fro flow signal between the aneurysm and the myocardial dissection, the systolic colorful shunts of the blocked outflow tracts, and severe aortic insufficiency (Fig 3).
5. In the case in which the aneurysm protruded into the interatrial septum, the aneurysmal body formed a saccular lesion in the interatrial septum and communicated with the NCS. The dark space in the septum expanded into the left and right atria, with no significant morphological change, movement, or compression of cardiac structure. CDFI showed the vortex flow in the saccular lesion of the septum (i.e. aneurysmal body) communicating with the aortic root.
6. In 2 cases, one in which the aneurysm ruptured into the LA and the other into the pulmonary artery, the echocardiographic features were not particularly distinct, similar to those extending into the right heart chambers except for the rare extending positions.
7. One aneurysm with a tortuous course had a prolonged thinner wall sac that extended into the RA near the tricuspid annulus. It then went through the orifice of the tricuspid valve and finally ruptured into the RV, causing tricuspid obstruction and insufficiency. The separatrix between the aneurysm and tricuspid septal cusp was not clear (the adhesion was confirmed by the operation).

## Discussion

The rare pathological patterns of congenital SVAs include a rare origin (from multiple sinuses or LCS), a rare extending position (same aneurysm into multiple cardiac chambers, left heart chamber, myocardium, pulmonary artery, superior vena cava, extracardiac position) and a tortuous course involving multiple cardiac structures. In our study, we found all of the rare patterns except for aneurysmal extension into the superior vena cava. The prevalence of the patterns among our group ranged from 0.4% to 3.3%, which was consistent with other reports [1,2,4]. However, some reports [5,6] have suggested that involvement of the interventricular septum is more common among Indian and African populations. Choudhary et al. [5] reported that the dissection into the interventricular septum occurred in 7.5% of 147 Indian patients with SVA.

Echocardiography is the modality of choice for the diagnosis of SVA. In over 95% of the patients with SVA at our hospital, surgical repair was undertaken based solely on the echocardiographic results with excellent diagnostic sensitivity (93.9%), specificity (99.9%), and accuracy (99.8%) [3]. However, in patients with SVA with rare patterns, special image characteristics that are noticeably different from those observed in the more common conditions were found because of the unique protrusion position, complex origin, tortuous course, or rare complication. Making a diagnosis is a challenge, especially for less experienced ultrasonographers. Therefore, to improve diagnostic accuracy, an experientially based comprehensive grasp of the criteria for ultrasonographic and differential diagnosis of these special SVA patterns is extremely important.

### Echocardiographic characteristics of different rare patterns

#### Aneurysm dissecting into the myocardium

When the SVA dissects into the interatrial septum, interventricular septum, or ventricular free wall, an echolucent space is visualized that is encapsulated by the separated myocardium, bulging into both sides and even forming a "third chamber" between the atria and the ventricles (Fig 3D). A flow signal filling it from the aortic root can be demonstrated with 2D and color Doppler echocardiography. The dark space is thought to be the aneurysmal body if the aneurysm is unruptured and undergoes inconspicuous morphological change during the cardiac cycle. CDFI generally detects the internal flow that is directly continuous with the aortic root. An aneurysmal thrombosis may occur in this condition. If the aneurysm ruptures, it is thought that the encapsulated blood resides in the muscular portion connecting the aneurysm. The morphological characteristics of the fluid area usually change, displaying a characteristic collapse during systole and expansion during diastole. The perforation of the aneurysm may not be easily detected at times and should be carefully evaluated from multiple planes. CDFI shows a to-and-fro signal at the site of perforation of the aneurysm, filling the dissected space from the aneurysm during diastole but reversing direction during systole.

Some complications should be given special attention, including obstruction of the left or right ventricular outflow tract due to the space-occupying effect of the myocardial dissection, thrombosis in the dissection lumen, calcification or fibrillation of the dissection intima, conduction disturbances (especially conduction block), and segmental ventricular wall motion abnormalities. In our cases, the only patient with an aneurysm dissecting into the interventricular septum suffered both left and right ventricular outflow tract obstructions, left bundle branch block, and severe aortic regurgitation.

The unruptured SVA protruding into the myocardium should be differentiated from a myocardial cyst. The echolucent cavity in a patient with SVA is filled with flow signals and is continuous with the aortic root. Its area is somewhat changeable during every cardiac cycle, though this is not obvious. The morphological characteristics of the fluid cavity of the myocardial cyst are stable: no communication to the aortic root and no blood flow. These qualities make them easy to differentiate. However, it should be noted that when the SVA dissection is small and has a thrombus, the morphological characteristics of the echolucent cavity might also be stable with no flow signal within it, similar to the myocardial cyst [7].

#### Aneurysm extending into LV

On the echocardiogram, a thin-walled saccular lesion arises at a low site between the sinus base and the aortic annulus and extends into the left ventricular outflow tract. The aneurysm usually goes back and forth between the aortic root and the left ventricular cavity with diastolic dilation and systolic compression [8]. This pattern is the only one of the ruptured SVA patterns that represents a single diastolic shunt (other patterns represent a continuous or dual-phase shunt). It is worth noting that of our 4 cases extending into the LV, the aneurysm went back and forth between the LV and RV via a huge ventricular septal defect in just one case. To our knowledge, this is the first report of this special representation in patients with SVA.

Compared with other patterns, the aneurysm originating from a low site of the sinus can more easily lead to aortic regurgitation. In addition to dilation of the annulus and valvular prolapse, secondary contributing factors may be hypoplasty of the adjacent cusp or prolapse of both cusps and annulus due to decreased support. Therefore, the morphology of the cusps and the motion of the annulus should be scrutinized to guide clinical decisions. Prolapse of the cusp and annulus was observed in all of our 4 cases with LV involvement and an adjacent cusp was found to be hypoplastic in 1 case. Complications in this pattern also include obstruction of the left ventricular outflow tract, thrombosis in the aneurysmal body, and myocardial ischemia. Myocardial ischemia may result from intermittent coronary ostial obstruction when the aneurysm moves into the aortic root during systole or coronary insufficiency by massive aortic regurgitation [9].

An aneurysm rupturing into the LV should also be differentiated from an aorto-left ventricular tunnel, an avulsed inner membrane of the aorta protruding into the left ventricular outflow tract during dissection of the aortic root, a ruptured perivalvular abscess, and severe prolapse of aortic valve. An aorto-left ventricular tunnel arises above the level of the ostia of the coronary arteries and consists of a tunnel or ampullar-like pattern without significant morphological change and movement [10]. In a patient with aortic dissection, in the presence of hypertension, the inner membrane protrudes into the LV not through the perivalvular location but through the valvular orifice without displacing the aortic annulus. A ruptured perivalvular abscess of the aortic valve is associated with typical echocardiographic findings of infective endocarditis such as vegetations. The abscess has a smaller area and a partially thin or thick wall. Its morphology and location are relatively stable. Severe prolapse of the aortic valve displays a flail-pattern movement of the involved cusp and stagger of the valvular tips without displacement of the aortic annulus.

#### Aneurysm extending into LA or pulmonary artery

In addition to their special extending positions, the echocardiographic features of these two patterns are similar to those extending into the right heart chambers. It should be noted that a huge aneurysmal body might obstruct the mitral valve [11] or pulmonary trunk.

A SVA extending into the LA should be differentiated from an annular subaortic aneurysm extending into the LA [12]. The SVA originates above the aortic annulus with communication to the aortic root and is dilated during diastole and compressed during systole. On the contrary, the annular subaortic aneurysm originates beneath the aortic annulus with communication to the left ventricular outflow tract and is dilated during systole and compressed during diastole.

#### Extracardiac SVA

An extracardiac SVA either protrudes from a higher region of the sinus of Valsalva than an intracardiac SVA or it diffusely involves the whole sinus, usually reported to be larger than the intracardiac sinuses because the limitations of pressure and volume are relatively smaller [13]. Echocardiography can detect the diffuse expansion of a thin-walled involved aortic sinus. The linear or shell-like enhanced and thickened wall can be visualized if it is calcified. The morphological changes and the mobility of the extracardiac aneurysms are not apparent during each cardiac cycle. In this pattern, a thrombus in the aneurysmal body is likely to occur and should be carefully evaluated. The huge aneurysm may distort the aortic annulus and root resulting in both aortic regurgitation and rotation of the aortic root. Therefore, the aneurysmal origin must be identified using only the short-axis view of the aortic root and not the long-axis view of the LV.

A huge extracardiac aneurysm can compress the adjacent cardiac structures (mainly the atrium or the affiliated venous structures) causing anatomical deformation and even obstruction, as seen in 2 of our cases. It may occasionally cause myocardial ischemia due to severe distortion of the coronary ostia or compression of the coronary trunk. The most acute and fatal complication of extracardiac SVA is spontaneous rupture into the pericardial or pleural cavity [14].

An extracardiac SVA involving all sinuses should be differentiated from an aneurysm of the ascending aorta involving the aortic sinuses. In patients with a SVA, the dilation of the aortic sinuses occurs prior to that of the ascending aorta and can compress the adjacent cardiac structures. Nevertheless, an aneurysm of the ascending aorta involving aortic sinuses might be a complication associated with Marfan's syndrome, atherosclerosis, or other diseases. Dilation of the aorta preferentially localizes in the ascending aorta and rarely causes the compression.

In our group, one extracardiac case from the LCS was misdiagnosed as a diverticulum of the LCS because it looked like a saccular bulge. However, an aortic root diverticulum is extremely rare. Our literature search uncovered only one report in a Chinese document that described a cecal tube-like structure with a small opening and a deep cavity arising from the aortic root at the valve commissure [15]. Except for reports describing a Kommerell diverticulum of the aortic arch, there are no other similar cases with which to compare experiences. We suggest that the sac structure should be diagnosed as a SVA if its opening is confined to an aortic sinus. An aortic root diverticulum should be considered if it appears at the sinus commissure with a “narrow neck and deep belly.”

#### Aneurysm extending into multiple chambers

In this pattern, the aneurysm usually originates near or at the tricuspid annulus and extends into both the RA and RV, as reported by our group. The sac may be observed fusing with the tricuspid valve to varying degrees, especially when associated with the vegetation. The fusion makes it difficult to judge the extending position, which led to a misdiagnosis because, in our cases, it only protruded into the RA. TEE may be helpful to precisely display the relationship between the aneurysm and the adjacent structures. Tricuspid insufficiency is the most common complication caused by the compression and displacement of the tricuspid valve.

#### Aneurysm arising from the LCS

Clinically, an aneurysm arising from the LCS can be found extending into the pulmonary artery or left atrium when arising from the base; into the left ventricle when arising from the portion between the sinus wall and the annulus; into the extracardiac position when arising from the higher region; and occasionally dissecting the interventricular septum or the interatrial septum [6,16]. In our group, the aneurysms arising from the LCS had an extracardiac protrusion in 1 case and ruptured into the LV in the other.

Determination of the origin of the SVA plays a key role in diagnosis. One can accurately assess its origin from the short-axis echocardiographic view of the aortic root; echocardiography is the diagnostic method of choice. Distinct echocardiographic features of different pathological patterns originating in the LCS have been discussed in the preceding sections according to the positions of their protrusions.

#### Aneurysm with tortuous course

A SVA usually extends directly into the adjacent cardiac chamber or vessel. In a few reports, the elongated aneurysmal body forms a tunnel and goes through several cardiac structures. The pathological anatomy and hemodynamic changes of these cases are extremely complex, especially when they occur concomitantly with other cardiovascular malformations. Choudhary et al. [4] reported one aneurysm from the LCS that formed a long tunnel and finally ruptured in the connection between the superior vena cava and the RA via the interatrial septum. Yildirim et al. [17] reported an aneurysm arising from the LCS and dissecting into the interatrial septum, forming a long tunnel and finally rupturing into the RA. In our cases, one aneurysm from the RCS extended into the RA, went through the tricuspid valve, and finally ruptured into the RV.

The complex and variable aneurysmal course combined with the possibility of multiple openings into several extending positions make echocardiographic diagnosis extremely challenging. Our case of a complex course was simply diagnosed as a SVA rupturing into the RV, and we did not detect the tortuous course because of lack of experience; the tortuous course was later identified during the operation. We suggest that once a long aneurysm forms a tunnel, this special pattern should be routinely investigated and excluded. The operator must carefully evaluate its origin and course using multiple views, including the non-standard planes.

### Combined diagnosis with other imaging techniques

Preoperative diagnosis of SVAs with rarely occurring patterns is most frequently made in Westerners in combination with an aortogram or a series of CT angiograms [4,16,17]. In contrast, the preoperative diagnosis in Chinese patients is based primarily on echocardiograms. In patients for whom the quality of the transthoracic images is poor, or for whom the diagnosis by transthoracic echocardiography is a challenge, TEE is strongly recommended. TEE can improve the diagnostic accuracy because of its better image quality, especially in the display of fine structures (e.g., a small aneurysmal defect, vegetations) and the involvement of multiple structures in the aneurysms originating from multiple sinuses, extending into multiple chambers, or having a tortuous course. Our results suggested that 3D echocardiography is superior to conventional ultrasound in the description of the morphological details of the aneurysm, in its relationship with the adjacent structures, and in identifying a small defect. Sometimes echocardiography is limited in its ability to highlight visually the spatial relationship between the aneurysm and its adjacent structures. CT angiography or aortic angiography is therefore usually recommended, especially with an extracardiac aneurysm or an aneurysm with a tortuous course.

## Conclusions

Different rare patterns of SVAs have distinct echocardiographic features and unique associated complications. Echocardiography is the diagnostic modality of choice in China. However, on occasion it may be difficult to delineate the origin and course of the aneurysm or precisely define the relationship of the aneurysm and adjacent structures with echocardiography alone. Therefore, a combination of echocardiography with other imaging techniques such as TEE, 3D echocardiography, CT angiography, and aortic angiography, is recommended to obtain more comprehensive information and to improve the diagnostic accuracy.

## Supporting information

S1 Checklist PLOS one clinical studies checklist. (DOCX)
