# Correction: Echocardiographic diagnosis of rare pathological patterns of sinus of Valsalva aneurysm

**DOI:** 10.1371/journal.pone.0197723

**Published:** 2018-05-16

**Authors:** Yali Yang, Li Zhang, Xinfang Wang, Qing Lü, Lin He, Jing Wang, Bin Wang, Ling Li, Li Yuan, Jinfeng Liu, Shuping Ge, Mingxing Xie

There are errors in the author affiliations. The affiliations should appear as shown here:

Yali Yang^1,2^, Li Zhang^1,2^, Xinfang Wang^1,2^, Qing Lü^1,2^, Lin He^1,2^, Jing Wang^1,2^, Bin Wang^1,2^, Ling Li^1,2^, Li Yuan^1,2^, Jinfeng Liu^1,2^, Shuping Ge^1,2,3^, Mingxing Xie^1,2^

**1** Department of Ultrasound, Union Hospital, Tongji Medical College, Huazhong University of Science and Technology, Wuhan 430022, China, **2** Hubei Province Key Laboratory of Molecular Imaging, Union Hospital, Tongji Medical College, Huazhong University of Science and Technology, Wuhan 430022, China, **3** The Heart Center, St. Christopher's Hospital for Children and Drexel University College of Medicine, Philadelphia, Pennsylvania, United States of America
